# Executive functions and borderline personality features in adolescents with major depressive disorder

**DOI:** 10.3389/fnhum.2023.957753

**Published:** 2023-06-22

**Authors:** Mona Albermann, Sophie Emery, Noemi Baumgartner, Michael Strumberger, Suzanne Erb, Lars Wöckel, Ulrich Müller-Knapp, Bruno Rhiner, Brigitte Contin-Waldvogel, Silke Bachmann, Klaus Schmeck, Gregor Berger, the Omega-3 Study Team, Noemi Baumgartner, Isabelle Häberling

**Affiliations:** Department of Child and Adolescent Psychiatry, University Hospital of Psychiatry, University of Zurich, Neumünsterallee 9, 8032 Zurich, Switzerland; Department of Child and Adolescent Psychiatry, University Hospital of Zurich; Department of Child and Adolescent Psychiatry, Psychiatric University Hospitals Basel; Child and Adolescent Psychiatric Services Baselland; Clienia Littenheid; Child and Adolescent Psychiatric Services Thurgau; Child and Adolescent Psychiatric Services St. Gallen; Klinik Sonnenhof; Department of Child and Adolescent Psychiatry, University Hospital of Zurich; University of Heidelberg; University of Basel; Epidemiology, Biostatistics, and Prevention Institute, University of Zürich; Clinical Trials Pharmacy (Kantonsapotheke) Zürich; ^1^Department of Child and Adolescent Psychiatry and Psychotherapy, University Hospital of Psychiatry Zurich, University of Zurich, Zürich, Switzerland; ^2^Psychiatric Hospital St. Gallen Nord, Wil, Switzerland; ^3^Research Department of Child and Adolescent Psychiatry, Psychiatric University Hospitals Basel, University of Basel, Basel, Switzerland; ^4^Child and Adolescent Psychiatric Services St. Gallen, St. Gallen, Switzerland; ^5^Clienia Littenheid AG, Littenheid, Switzerland; ^6^Child and Adolescent Psychiatry Klinik Sonnenhof, Ganterschwil, Switzerland; ^7^Child and Adolescent Psychiatry Thurgau, Weinfelden, Switzerland; ^8^Child and Adolescent Psychiatric Services Baselland, Basel, Switzerland; ^9^University Clinic of the Martin-Luther University Halle – Wittenberg’s Medical Faculty, Halle, Germany; ^10^Département de Psychiatrie, Hôpitaux Universitaires de Genève, Geneva, Switzerland

**Keywords:** executive function, major depressive disorder, borderline personality disorder, adolescents, emotion regulation, inhibition

## Abstract

**Background:**

Executive functions (EF) consolidate during adolescence and are impaired in various emerging psychiatric disorders, such as pediatric Major Depressive Disorder (pMDD) and Borderline Personality Disorder. Previous studies point to a marked heterogeneity of deficits in EF in pMDD. We examined the hypothesis that deficits in EF in adolescents with pMDD might be related to comorbid Borderline Personality features (BPF).

**Methods:**

We examined a sample of 144 adolescents (15.86 ± 1.32) diagnosed with pMDD. Parents rated their child’s EF in everyday life with the Behavior Rating Inventory of Executive Function (BRIEF) and BPF with the Impulsivity and Emotion Dysregulation Scale (IED-27). The adolescents completed equivalent self-rating measures. Self- and parent-ratings of the BRIEF scores were compared with paired t-Tests. Correlation and parallel mediation analyses, ICC, and multiple regression analyses were used to assess symptom overlap, parent-child agreement, and the influence of depression severity.

**Results:**

Over the whole sample, none of the self- or parent-rated BRIEF scales reached a mean score above T > 65, which would indicate clinically impaired functioning. Adolescents tended to report higher impairment in EF than their parents. Depression severity was the strongest predictor for BPF scores, with *Emotional Control* predicting parent-rated BPF and *Inhibit* predicting self-rated BPF. Furthermore, the Behavioral Regulation Index, which includes EF closely related to behavioral control, significantly mediated the relationship between depression severity and IED-27 factors *emotional dysregulation* and *relationship difficulties* but not *non-suicidal self-injuries.*

**Conclusion:**

On average, adolescents with depression show only subtle deficits in executive functioning. However, increased EF deficits are associated with the occurrence of comorbid borderline personality features, contributing to a more severe overall psychopathology. Therefore, training of executive functioning might have a positive effect on psychosocial functioning in severely depressed adolescents, as it might also improve comorbid BPF.

**Clinical trial registration:**

www.ClinicalTrials.gov, identifier NCT03167307.

## Introduction

Adolescence as a period of transition is characterized by structural changes and a reorganization of brain functions, creating an imbalance between earlier maturing areas associated with the reward system and emotion processing and later maturing prefrontal areas associated with cognitive control (dual systems model) (Gogtay et al., 2004; Casey et al., 2008; Konrad et al., 2013; Luciana, 2013; Blackmore and Mills, 2014; Mills et al., 2015). Cognitive control is enabled by executive functions (EF), mainly in the prefrontal cortex (Diamond and Lee, 2011). EF include cognitive flexibility, inhibition (self-control and self-regulation), working memory, problem-solving, and planning (Miyake et al., 2000; Drechsler, 2007; Gürdere et al., 2023). An imbalance of these regulatory functions may lead to behaviors such as enhanced risk-taking or impulsive decision-making, often observed in teenagers. However, it may also increase the vulnerability to psychological distress, negative social environments, or peer adversity, which may in turn contribute to the development of psychopathology in this critical period of life (Luciana, 2016). Given that deficits in EF in adolescents have been described as a general risk marker for psychopathology, such as paediatric depression (Fenesy and Lee, 2019; Romer and Pizzagalli, 2021), the early identification of EF impairment is highly relevant for clinical practice, considering that in adolescents training can improve EF (Carr and Stewart, 2019; Pasqualotto et al., 2021).

Pediatric Major Depressive Disorder (pMDD) is among the most frequent psychopathological disorders in adolescents, with an estimated 12-month prevalence of 7.5% and a lifetime prevalence of 11% (Avenevoli et al., 2015; Polanczyk et al., 2015). Borderline Personality Features (BPF) and Borderline Personality Disorder (BPD) are frequent comorbidities of depression. More than half of the adolescents with a BPD diagnosis present a co-occurring pMDD diagnosis (Pham-Scottez, 2016). In adults, about half of the individuals with BPD meet the criteria for MDD, while 10–30% of individuals with MDD have co-occurring BPD (Rao and Broadbear, 2019). BPD is considered a disorder in its own right and not as a variant of either MDD or bipolar affective disorder (Beatson and Rao, 2012). Dysfunctional emotional regulation is a key feature (Chapman, 2019), other characteristics are inconsistent identity, and unstable interpersonal relations (Lieb et al., 2004; Bohus et al., 2021). According to DSM-5, at least five out of nine of the following features must be present for a diagnosis of BPD: fear of abandonment, unstable relationships, unstable self-image, impulsivity, self-harm, mood instability, feelings of emptiness, inappropriate anger, and dissociation/transient paranoid ideation (DSM-5 APA; see Bohus et al., 2021). For diagnosis in adolescence, symptoms need to persist for at least one year. In the past, personality disorders in adolescents have been underdiagnosed, as clinicians and researchers have been hesitant to apply the concept of personality disorders to children and adolescents in part to prevent pathologization and stigmatization (Kaess et al., 2020). At this point, there is sufficient evidence in favor of a BPD diagnosis in adolescents as it has major implications for treatment planning (Miller et al., 2008; Kaess et al., 2014). Prevalence estimates in adolescents vary between 1.4% (Johnson et al., 2008) and 6.3% (Guilé et al., 2021) in the population; but are much higher in clinical samples (11–50%; see Kaess et al., 2014).

Both BPD and pMDD have been explained using the biopsychosocial model, with a combination of genetic factors, personality traits, and adverse events during childhood as underlying factors (Lieb et al., 2004; Chapman, 2019). On a neuroanatomical level, BPD has been associated with volume reductions in the amygdala, the hippocampus, the orbitofrontal cortex (OFC), the frontal lobes, and the cingulate cortex in adults, and with OFC volume reduction in adolescents (Chanen et al., 2008). Particularly the amygdala has been associated with the regulation of negative affective stimuli, which is relevant for regulatory control (Soloff et al., 2017). fMRI studies have shown a hyper-arousal of the amygdala in patients with BPD, which lead to the projection of negative attributes onto neutral faces (Donegan et al., 2003). These processes of abnormal brain maturation may result in the characteristic features of BPD of emotional dysregulation and impulsivity in adolescents (Houston et al., 2005).

### Executive functions in adolescent with pMDD and BPD

Performance deficits in EF have been observed both in pMDD and BPD. A meta-analysis by Wagner et al. (2015) based on 33 studies analysing cognitive functions in youth with depression provided evidence of EF deficits in the domains of *inhibition, verbal fluency, working memory, cognitive flexibility*, and *planning*, although findings in this regard are not consistent (Vilgis et al., 2015; Han et al., 2016). Evidence regarding EF deficits in BPD is also mixed, with several studies reporting problems in *inhibition, planning, cognitive flexibility*, and *working memory* (e.g. Hagenhoff et al., 2013), and others failing to find impairment in these domains or only in association with BPD subtypes or comorbidity (see McClure et al., 2016). Wante et al. (2017) showed a mediating effect of maladaptive and adaptive emotion regulation (ER) strategies on the association between EF impairment and depressive symptoms in adolescents. The greater the EF impairment the more maladaptive ER strategies were reported.

### Assessment of executive functions in everyday life

Multi-informant rating scales, such as the Behavior Rating Inventory of Executive Function (BRIEF; Gioia et al., 2000, 2002) are used to assess deficits in EF in everyday life behavior. While the clinical validity of the BRIEF has been demonstrated in a large number of studies in samples with neurological, developmental, psychopathological, or somatic disorders (McCandless and O’Laughlin, 2007; Drechsler et al., 2015; de Vries et al., 2018), the association between deficits in EF assessed by rating scales or by objective EF tests, which purportedly measure the same underlying EF construct, is often low (e.g. Toplak et al., 2010; Tran et al., 2021). While this does not call the validity of either method into question, one must keep in mind that scale-based and performance test-based EF measures provide complementary rather than equal information.

The BRIEF has rarely been used in adolescents with major depressive disorder (pMDD) as the primary diagnosis, but BRIEF indices have been shown to lie above the clinical threshold (>T65) in untreated children and adolescents with mood disorders (Vesco et al., 2018). In the adult version of the BRIEF (BRIEF-A, Roth et al., 2005), young adult patients with first-episode MDD indicated significantly higher deficits in EF compared to controls, with the largest effect sizes on *Task Monitor, Plan/Organize, Initiate, and Working Memory* (Schmid and Hammar, 2021).

A recent study compared parent-rated BRIEF profiles in adolescents with BPD of both the externalizing and the internalizing subgroups and found substantially higher impairment in the externalizing subgroup on all EF except for *Shift, Emotional Control, and Initiate*. Even in the internalizing subgroups though, the scale scores were above T60, indicating that the EF were impaired to a certain degree (Kalpakci et al., 2018). The authors concluded that one reason for the inconsistent findings on deficits in EF in BPD might be that the impact of the possible BPD subtypes has not been sufficiently considered in research, with EF apparently being particularly affected in the externalizing BPD subtype.

Taken together, deficits in EF have been reported in pMDD and in BPD, but no study to date investigated how EF are affected in depressed adolescents with comorbid borderline personality features.

### Agreement between parent- and self-report

In general, the agreement between self-reports and informant ratings on clinical impairment scales is often low to moderate at best, and concordance is affected by age, gender, and the nature of the impairment (van der Ende et al., 2012; De Los Reyes et al., 2015). Poor parent-child interrater agreement has been associated with poorer treatment outcome (Goolsby et al., 2018). The agreement is usually lower for internalizing disorders than for externalizing disorders, which has been attributed to the low observability of internalizing symptoms (see Vierhaus et al., 2018). Discrepancies between parents’ ratings and self-reports in adolescents with depression have been frequently observed (e.g. Kazdin et al., 1983; Baumgartner et al., 2020). The question whether adolescents or their parents tend to report more severe depression symptoms is unresolved; it has been claimed that in community samples, adolescents often report more severe depressive symptoms than do their parents (e.g. Eg et al., 2018; Stein et al., 2018), while the reverse can be found in clinical samples (see Makol and Polo, 2017), although the findings in this regard are not consistent. Interrater agreement for BPF in adolescents has rarely been analyzed, but Schuppert and colleagues reported poor informant agreement in a BPD interview, with parents reporting fewer symptoms than patients (Schuppert et al., 2012).

In depression, self-perception may be negatively biased, which may lead to an overestimation of cognitive deficits and other symptoms (e.g. Schwert et al., 2018; Serra-Blasco et al., 2019). Negative self-evaluations are also characteristic of BPD (Winter et al., 2017). Concerning ratings of EF, self-reports may also be biased because of cognitive impairment and diminished awareness (e.g. Krasny-Pacini et al., 2015). However, in individuals with relatively mild cognitive deficits, awareness of cognitive problems encountered in everyday life may be enhanced. At the same time, relatives may not notice these difficulties, which the affected person may try to compensate for or to dissimulate (e.g. Rizzo et al., 2012; Puhr et al., 2019). Parent ratings of depression and/or BPD symptoms may also be biased, e.g. underestimating, misidentifying, or – rarely – overestimating depressive symptoms in their teenage child (Madjar et al., 2020). Parents may be unaware of their child’s inner conflicts or suicidal thoughts (Jones et al., 2019).

This present study investigated EF in everyday life of adolescents with pMDD and analyzed the possible impact of deficits in EF on BPF tendencies in this group. The following research hypotheses and questions guided our analyses:

– We expected adolescents with pMDD to have deficits in EF, especially in the scales *Monitor, Plan/Organize, Initiate*, and *Working Memory.*
– We expected adolescents to report greater deficits on the BRIEF self-rating scales compared to their parents’ report on the BRIEF parent-rating scales, thus reflecting poor interrater agreement.
– We hypothesized that pMDD patients with more severe EF impairment and greater depression severity would show elevated BPF. In particular, we expected that more severe deficits on BRIEF scales *Inhibit, Emotional Control*, and *Monitor*, the three domains directly related to BPD core features, would predict higher BPF scores. We also sought to investigate the possible overlap between the concepts of BPF and EF.
– We hypothesized that deficits in EF would mediate the association between depression severity and BPF, especially for the BRIEF *Behavioural Regulation Index*, as it includes the *Inhibit* and *Emotional Control* scales.

## Materials and methods

### Recruitment, participants, and procedure

The data used for this analysis were gathered at the baseline visits of the omega-3-pMDD study of the University of Zurich (Switzerland). The main goal of the study is to assess the efficacy and safety of omega-3 fatty acids in the early course of paediatric major depressive disorder (pMDD) (Häberling et al., 2019). Inclusion criterion was a major depressive disorder according to DSM-IV (APA, 1994) with at least moderate symptom severity (cut-off score of the Children’s Depression Rating Scale-Revised (CDRS-R) ≥ 40). Exclusion criteria were pre-existing neurological disorders, lifetime diagnosis of schizophrenia or bipolar affective disorder, pervasive developmental disorder, severe conduct disorder, intellectual disability, substance dependency, but not misuse (ICD-10 F1x.2) or eating disorders (ICD-10 F 50.0 and 50.2) within the last six months. Additional inclusion criteria for the present study were age 13 to 17 years and complete data sets for the relevant instruments (BRIEF self-rating (SR), BRIEF parent-rating (P), Scale of Impulsivity and Emotion Dysregulation self-rating (IED-27 SR), Scale of Impulsivity and Emotion Dysregulation parent-rating (IED-27 P)).

Recruitment took place in various inpatient and outpatient units of seven departments of child and adolescent psychiatry in the German speaking part of Switzerland. The data were collected before randomization to one of the two treatment arms. The participants were visited by trained study staff either in the psychiatric hospital or at home. Patients and parents gave informed written consent before entering the study. The study was approved by the local ethics committees (www.ClinicalTrials.gov, identifier NCT03167307).

A total of 310 children and adolescents were screened, and 257 were randomized. After data cleaning, the sample for the present study consisted of 144 parent-child dyads. The adolescents’ mean age was 15.86 years (SD = 1.32), and 74.3% of the sample were female.

### Measures

#### Executive functions

Deficits of EF in everyday life were assessed using the German version of the BRIEF (Gioia et al., 2002; Drechsler and Steinhausen, 2013), as briefly described in the introduction. The original BRIEF structure was supposed to have a two-factor structure, which is reflected by two overarching indices: the *Behavioural Regulation Index* (*BRI*) with its scales *Inhibition, Shift, Emotional Control*, and the *Metacognition Index* (*MI*), comprising the scales *Working Memory, Initiate, Monitor, Plan/Organize*, and *Organization of Materials*. However, the initial two-factor structure has been questioned by various studies (e.g. Halvorsen et al., 2019). In 2015, a shorter form, the BRIEF-2 (Behaviour Rating Inventory of Executive Function, Second Edition; Gioia et al., 2015) was published, which is based on a three-factor structure, reflected by three indices: the *Behavior Regulation Index* (*BRI*), the *Emotion Regulation Index* (*ERI*), and the *Cognitive Regulation Index* (*CRI*). In the present paper, the original BRIEF scales are used. For comparison, additional analyses based on the BRIEF-2 scale structure can be found in Supplementary Tables 3, 4.

#### Borderline personality features

Borderline personality features (BPF) were assessed using the Scale of Impulsivity and Emotion Dysregulation (IED-27-J) in its adapted version for children and adolescents (Kröger and Kosfelder, 2011; Kröger et al., 2017; Dreysse et al., 2021). The IED-27-J is a 27-item questionnaire rated by children and their parents (see Supplementary Table 1 for self-rating items and Supplementary Table 2 for parents’ items). Borderline specific experiences and behavioral tendencies during the past month are to be rated on a 5-point Likert-scale (“never”, “1-2 times”, “3-10 times”, “daily”, “multiple times daily”) by the adolescents themselves and on a 3-point Likert-scale by the parents. While the scale was originally been developed for adults, the adaptation for adolescents has demonstrated good validity and rel iability (Kröger et al., 2017). Factorial analysis of the adult’s version resulted in three main factors: *emotional dysregulation, relationship difficulties*, and *self-injuries and suicidal behavior* (Dreysse et al., 2021).

#### Clinical assessment and IQ

Diagnosis of pMDD and other possible comorbid psychopathological disorders were based on the German version (Delmo et al., 2001) of the diagnostic interview Kiddie Schedule for Affective Disorders and Schizophrenia for School-Age Children (K-SADS; Kaufman et al., 1997), and depression severity was rated using the German version of the Children’s Depression Rating Scale – Revised (CDRS-R) (Poznanski et al., 1996; Keller et al., 2011, 2012). Both assessments are based on the clinician’s evaluation of the combined interviews of the adolescent and his or her parents. The interviews are conducted with the child and the parent separately and the trained clinician then integrates the parents’ and children’s reports to reach a final score. The 17 items of the CDRS-R quantify depressive symptoms over the past two weeks. A total score below 30 indicates no diagnosis of depression, 30–40 a mild depressive episode, and 40–60 a moderate depressive episode, and ≥ 60 a severe depressive episode. The maximum possible score is 113. The scale has been extensively used in research (Häberling et al., 2019). The IQ was assessed using the German adaptation (Hagmann-von Arx and Grob, 2014) of the Reynolds Intellectual Scales (RIAS; Reynolds and Kamphaus, 2003).

### Statistical analysis

For the BRIEF scales, either raw scores or age- and gender adjusted T-values were used. BRIEF-T-values were used for all analyses except when analysing the conceptual overlap of EF and BPF using simple correlations, as described below (Drechsler and Steinhausen, 2013). Generally, a T-value above 65 indicates a clinical impairment, with T-values above 60 indicating subclinical deficits. For comparisons between parent-rating (P) and self-rating (SR), the BRIEF P *Self-Monitor* subscale was compared with the BRIEF SR *Monitor* scale because of the respective item structure. The comparison of self-rated and parent-rated EF was calculated with t-tests for paired samples with Bonferroni correction for multiple comparisons applied.

Agreement between parent and child ratings on the BRIEF scales was analyzed with the intraclass correlation coefficient (ICC) (McGraw and Wong, 1996; Koo and Li, 2016), which is a standard reliability index. According to Koo and Li (2016), based on the 95% confidence interval of the ICC estimate, values below 0.5 indicate poor reliability and values between 0.5 and 0.75 indicate moderate reliability. The possible overlap of the concepts of EF and BPF was analyzed based on correlations of the BRIEF SR and BRIEF P scales (raw-scores) with the three factors structure of the IED-27 SR and IED-27 P proposed by Dreysse et al. (2021), applying Bonferroni correction to control for multiple comparisons.

To investigate the relationship between depression severity, EF and BPF, we calculated two multiple regression analyses: 1. self-rated IED-27 total score as dependent variable with self-rated BRIEF scales and CDRS total score as independent variables; 2. parent-rated IED-27 total score as dependent variables with parent-rated BRIEF scales and CDRS total scores as independent variables (pre-requirements were met).

To examine whether EF mediate the relationship between depression severity and BPF. we conducted three parallel mediation models using model 4 of the SPSS PROCESS macro by Hayes (2018). As the CDRS includes items about suicidal behavior and suicidal thoughts, we extracted four factors of the IED-27 SR: *emotional dysregulation, relationship difficulties, suicidal behavior*, and *non-suicidal self-injuries (nssi)* (based on Dreysse et al. (2020)). The factor *suicidal behaviour* was not included in any analysis. Three different mediation models were calculated, with *BRI* and *MI* of the BRIEF SR as parallel mediators of the relationship between depression severity and the three IED-27 SR factors *emotional dysregulation*, *relationship difficulties*, and *nssi*. The CDRS score used in the analysis was based on the child’s assessment of items 1–14, as ratings of items 15–17 are based solely on the clinician’s perception. As covariates, we included age, gender, and IQ. Process uses a standard bootstrapping approach based on 5,000 samples that provides confidence intervals for indirect effects. Confidence intervals that do not include 0 provide evidence for a statistically significant mediation effect. Statistical analyses were conducted using SPSS Version 27 for Windows and Version 28 for Mac (IBM Corp, 2020, 2021), Excel (Microsoft Excel, 2018), and R Version 2022.07.2+576 for Mac (R Core Team, 2022).

## Results

### Sample description

Descriptive statistics of the sample is listed in Table 1. The adolescents in our sample reported a mean IED-27 SR score of *M* = 34.03 (*SD* = 16.56; Min = 2, Max = 82). Parents reported a mean IED-27 P score of *M* = 20.06 (*SD* = 9.39; Min = 1, Max = 47). The mean depression severity score of our sample was *M* = 58.88 (*SD* = 8.39; Min = 42, Max = 85). The mean IQ was *M* = 104.46 (*SD* = 8.89; Min = 76, Max = 127). 67 adolescents reported the intake of antidepressants.

**TABLE 1:** Sample description.

	Total *N* = 144 (107 female/37 male)			
	*M* (SD)	Min		Max
Age	15.86 (1.32)	13.00		18.00
IED 27 SR	34.03 (16.56)	2		82
IED 27 P	20.06 (9.39)	1		47
CDRS tot	58.88 (8.39)	42		85
IQ	104.46^a^ (8.89)	76		127
**Antidepressants**	**Yes (n = 67)/No (n = 77)**			
	***N***			
pMDD			144	
*Comorbidities*				
Psychotic attributes			7	
AD(H)D			15	
Panic disorder			5	
Separation anxiety disorder			1	
Simple phobia			14	
Social phobia			11	
Agoraphobia			1	
Generalized anxiety disorder			16	
Obsessive-compulsive disorder			2	
Post-traumatic stress disorder			6	
Oppositional disorder			1	
Other			2	

CDRS tot = Children’s Depression Rating Scale – Revised total score. IED-27 = Scale of Impulsivity and Emotion Dysregulation. SR = self-rating; P = parent-rating; tot = total score; M = Mean; SD = Standard Deviation. ^*a*^*n* = 130.

### Comparison of self-rated and parent-rated EF (BRIEF scales)

**FIGURE 1:**
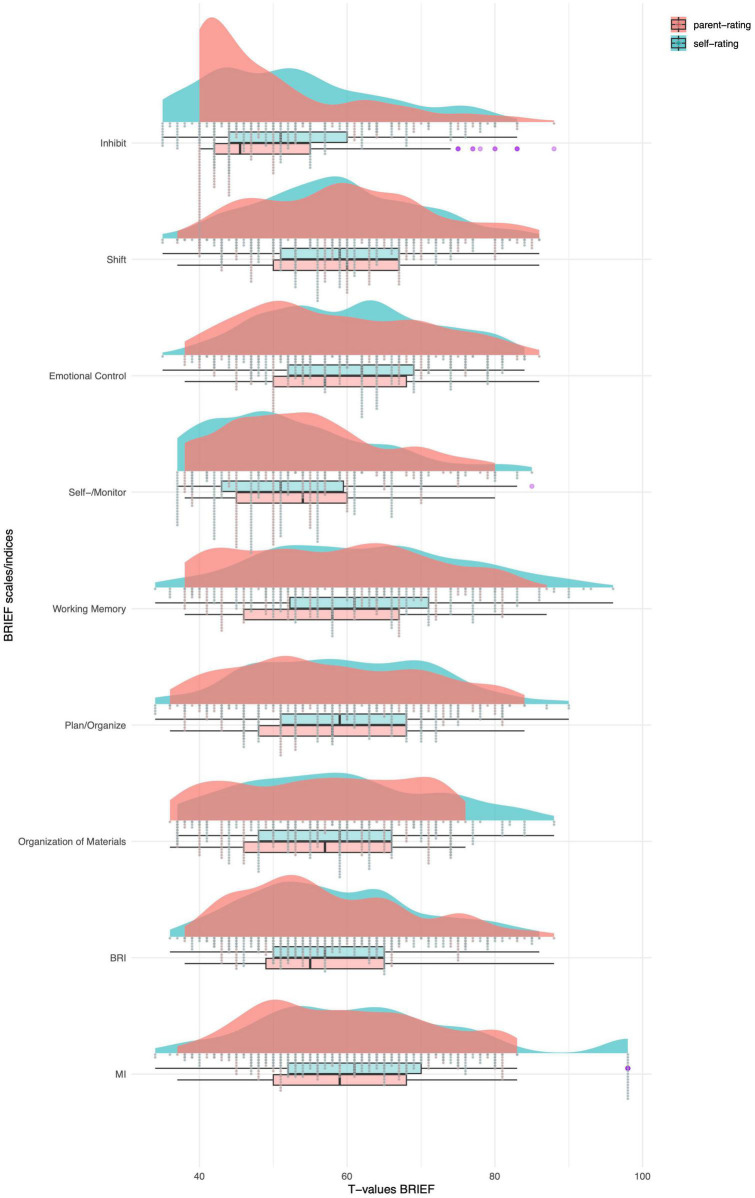
Means of self-rated and parent-rated BRIEF scales – T-values, *N* = 144. Monitor self-rating and self-monitor parent-rating scale. BRI = Behavior Regulation Index; MI = Metacognition Index; Purple = outliers.

Only the BRIEF SR scales *Emotional Control* (*M* = 61.10, *SD* = 10.80), *Working Memory* (*M* = 63.60, *SD* = 13.37), and *Plan/Organize* (*M* = 60.60 *SD* = 12.34) and the index *BRI* (*M* = 63.66, *SD* = 11.31) were rated above T > 60 by the adolescents (Table 2). None of the parent-rated BRIEF P scales scored above T > 60. The paired sample t-tests showed a significant differences between self-rating and parent-rating for the BRIEF scales *Inhibit* (*t* = 3.228, *p* < 0.001), *Emotional Control* (*t* = 3.314, *p* = 0.011), *Working Memory* (*t* = 4.898, *p* < 0.001), *Plan/Organize* (*t* = 2.948, *p* = 0.002), *Organization of Materials* (*t* = 2.711, *p* = 0.004) and the Index *MI* (*t* = 3.365, *p* < 0.001), with medium effect sizes (*d*) (Cohen, 1988). After Bonferroni correction for multiple comparisons (0.05/9 = 0.006), the rating difference for *Emotional Control* was no longer significant (Table 2 and Figure 1). Agreement between self-and parent-rated BRIEF scales was poor (<0.50) for all scales and indices.

**TABLE 2:** Total sample: t-Test comparison of BRIEF-SR and BRIEF-P scales and intraclass correlations – T-values.

BRIEF scales	Self-rating (*n* = 144)	Parent-rating (*n* = 144)	t-test comparison	Cohen’s *d*	ICC	*r*
	*M (SD)*	*M (SD)*				
Inhibit	53.38 (11.84)	49.78 (11.00)	*t*(143) = 3.228, *p* < 0.001	0.269	0.305**	0.319
Shift	58.90 (10.72)	59.02 (11.54)	*t*(143) = -0.103, *p* = 0.459	0.009	0.153*	0.152
Emotional Control	61.10 (10.80)	58.40 (11.85)	*t*(143) = 2.314, *p* = 0.011	0.193	0.235^mc^	0.242
Self-/Monitor^a^	52.73 (11.27)	54.34 (9.71)	*t*(143) = −1.524, *p* = 0.065	0.127	0.287**	0.291
Working Memory	63.60 (13.37)	57.40 (12.73)	*t*(143) = 4.898, *p* < 0.001	0.408	0.290**	0.322
Plan/Organize	60.60 (12.34)	56.75 (12.68)	*t*(143) = 2.948, *p* = 0.002	0.246	0.206^mc^	0.215
Organization of Materials	59.35 (13.32)	56.38 (11.24)	*t*(143) = 2.711, *p* = 0.004	0.226	0.419**	0.436
BRI	58.59 (11.31)	56.83 (11.02)	*t*(143) = 1.542, *p* = 0.063	0.129	0.242*	0.244
MI	63.66 (15.45)	59.03 (11.46)	*t*(143) = 3.365, *p* < 0.001	0.280	0.249**	0.274

*N* = 144. *M* = Mean; SD = Standard Deviation; ICC = Intra Class Correlation Coefficient (two-way random, absolute agreement, single measure). *r* = Inter Item Correlation (Pearson). *p* = *p*-value, one-sided, significance testing. Cohen’s *d* = effect size (0.2 = small, 0.5 = medium, 0.8 = large). BRI = Behavior Regulation Index; MI = Metacognition Index.

^*a*^Monitor SR scale and Self-Monitor P scale.

**p < 0.001, *p < 0.05.

^*mc*^*p*< 0.006 [Bonferroni correction for multiple comparisons for the t-tests: (0.05/9 = 0.006)].

#### Association between EF [BRIEF scales and BPF (IED-27 factors)]

We performed a correlation analysis to test for a possible overlap between BRIEF scales and IED-27 factors (Table 3). The *emotional dysregulation* factor correlated only weakly with the BRIEF scales and indices. The *relationship difficulties* factor, however, correlated highly with the parent-rated *Emotional Control* (*r* = 0.561, *p* < 0.001) scale and the index *BRI* (*r* = 0.548, *p* < 0.001). For the self-rated version, the *relationship difficulties* factor correlated highly with the scales *Inhibit* (*r* = 0.595, *p* < 0.001), *Emotional Control* (*r* = 0.624, *p* < 0.001) and the *BRI* (*r* = 0.659, *p* < 0.001). The *self-injuries and suicidal behavior* factor, on the other hand, did not correlate with any of the self-or parent-rated BRIEF scales or indices. A complete overview of the correlations between individual IED-27 SR/P items and the BRIEF scales can be found in the Supplementary Table 1 (self-rating) and Supplementary Table 2 (parent-rating). In the supplement Supplementary Tables 3, 4 the correlations between the BRIEF 2 subscales and the three IED-27 factors are listed, revealing a very similar pattern of correlation to the original BRIEF scales presented here.

**TABLE 3:** Correlations between the three IED-27 factors and the BRIEF scales and indices (raw scores).

BRIEF scales	IED-27 factors							
	Emotional dysregulation		Relationship difficulties		Self-injuries and suicidal behavior		Depression severity	
	SR	P	SR	P	SR	P	SR	P
Inhibit	0.373***	0.092	0.595***	0.363***	0.092	0.111	0.140	0.060
Shift	0.287***	0.234**	0.393***	0.374***	0.030	0.107	0.171*	0.201*
Emotional control	0.407***	0.332***	0.624***	0.561***	0.125	0.180	0.259^mc^	0.284***
Initiate (P)		0.175**		0.314***		0.126		0.349***
Working memory	0.249^mc^	0.203**	0.340***	0.144	0.138	0.109	0.248^mc^	0.300***
Plan/Organize	0.294***	0.161	0.388***	0.282***	0.102	0.089	0.194**	0.269^mc^
Organization of materials	0.271***	0.243^mc^	0.403***	0.079	0.104	0.186	0.225^mc^	0.015
Task-completion (SR)	0.206*		0.262**		−0.014		0.162	
Monitor	0.306***	0.104	0.440***	0.261^mc^	0.143	0.093	0.254^mc^	0.182*
Self-monitor (P)		0.083		0.242^mc^		−0.013		0.111
BRI	0.436***	0.282***	0.659***	0.548***	0.088	0.169	0.288***	0.234**
MI	0.289***	0.219**	0.392***	0.265^mc^	0.074	0.140	0.247^mc^	0.283***

IED-27 factors derived from self-ratings were correlated with self-rated BRIEF scales and IED-27 factors derived from the parent-rated IED-27 questionnaire were correlated with parent-rated BRIEF scales.

*N* = 144, P = parent-rating; SR = self-rating. Depression severity = CDRS score. Bold = *r* > 0.50. IED-27 factors from Dreysse et al. (2021). BRI = Behavior Regulation Index; MI = Metacognition Index.

^*mc*^*p* < 0.005 (corrected for multiple comparisons: 0.05/11).

***p < 0.001. **p < 0.01. *p < 0.05.

#### EF deficits and depression severity as predictors for BPF

To test the association between deficits in EF and BPF, we conducted two separate multiple regression analyses, as shown in Table 4. For the self-rating, the overall model was significant *F*(8,135) = 9.774, *p* < 0.001 with an adjusted *R*^2^ = 0.329. The CDRS total score (*b* = 0.729, *p* < 0.001) and the BRIEF scale *Inhibit* (*b* = 0.330, *p* < 0.033) significantly predicted the self-rated IED score. For the parent ratings, the model was also significant (*F*(8,135) = 7.214, *p* < 0.001) with an adjusted *R*^2^ = 0.258. The BRIEF scale *Emotional Control* (*b* = 0.173, *p* = 0.032) and the CDRS total score (*b* = 0.364, *p* < 0.001) significantly predicted the parent-rated IED total score.

**TABLE 4:** Multiple regression analyses for IED-27 total score with BRIEF scales and CDRS total score – parent-rating and self-rating – T-values.

Variables	Influence on IED-27 total score parent-rating			Influence on IED-27 total score self-rating		
	*B*	β	SE	*b*	β	SE
Constant	−27.164***			−45.397***		
Inhibit	0.114	0.133	0.084	0.330*	0.236*	0.153
Shift	0.103	0.127	0.081	−0.078	−0.050	0.145
Emotional control	0.173*	0.219*	0.080	0.185	0.121	0.148
Working memory	0.047	0.063	0.082	−0.149	−0.120	0.150
Plan/organize	−0.046	−0.063	0.087	0.179	0.133	0.160
Organization of materials	0.129	0.155	0.067	0.120	0.097	0.129
Monitor	−0.063	−0.067	0.095	0.070	0.047	0.130
CDRS total score	0.364***	0.325***	0.083	0.729***	0.369***	0.145
	*R* ^2^	0299		*R* ^2^	0.367	
	Corr. *R*^2^	0.258		Corr. *R*^2^	0.329	
	*F*(df = 8, 135)	7.214***		*F*(df = 8, 135)	9.774***	

*N* = 144. *b* = unstandardized regression coefficient. β = standardized regression coefficient. SE = standard error. *p < 0.05. ***p < 0.001.

#### Parallel mediation models – does EF impairment mediate the relationship between depression severity and borderline personality features?

**FIGURE 2:**
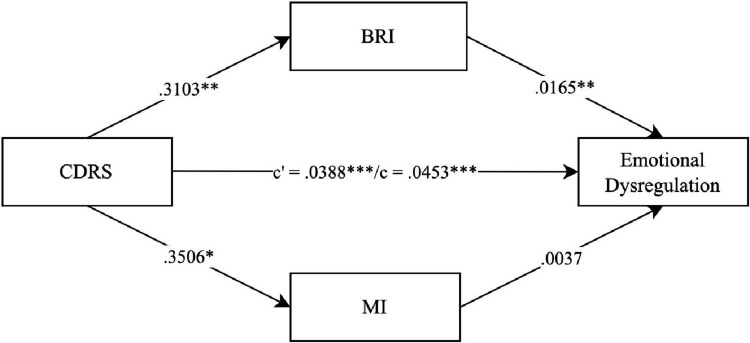
Parallel mediation model – CDRS, IED-27 SR factor emotional dysregulation. *N* = 144. CDRS score = items 1–14 child rating. BRI = Behavior Regulation Index; MI = Metacognition Index; BRIEF SR T-values. *c*’ = direct effect. *c* = total effect. ***p < 0.001. **p < 0.01. *p < 0.05. Covariates gender, age and IQ were included in the mediation, not shown due to non-significance.

**FIGURE 3:**
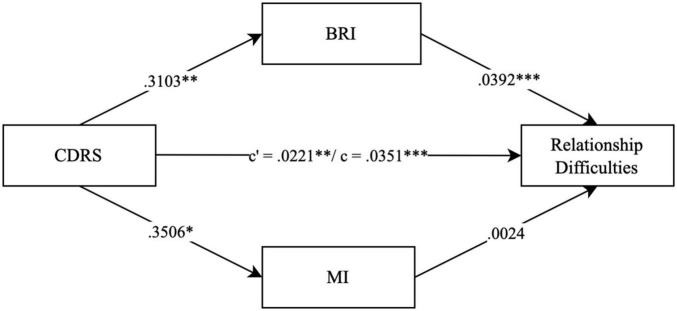
Parallel mediation model – CDRS, IED-27 SR factor relationship difficulties. *N* = 144. CDRS score = items 1-14 child rating. BRI = Behavior Regulation Index; MI = Metacognition Index; BRIEF SR T-values. *c*’ = direct effect. *c* = total effect. ***p < 0.001. **p < 0.01. *p < 0.05. Covariates gender, age and IQ were included in the mediation, not shown due to non-significance.

**FIGURE 4:**
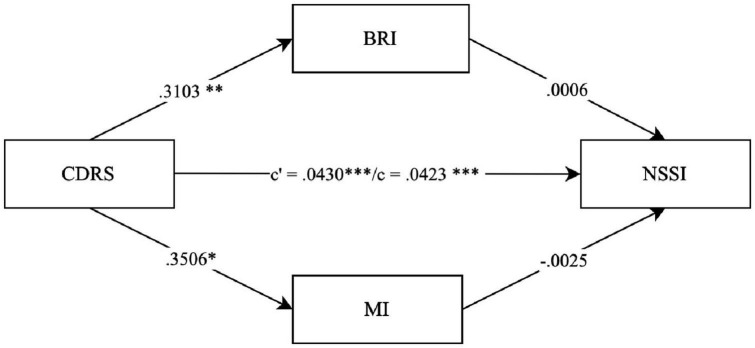
Parallel mediation model – CDRS, IED-27 SR factor nssi. *N* = 144. CDRS score = items 1-14 child rating. BRI = Behavior Regulation Index; MI = Metacognition Index; BRIEF SR T-values. *c*’ = direct effect. *c* = total effect. ***p < 0.001. **p < 0.01. *p < 0.05. Covariates gender, age and IQ were included in the mediation, not shown due to non-significance.

To further investigate the relationship between depression severity EF impairment and borderline personality features, we conducted three parallel mediation models in which EF impairment (BRIEF SR indices *BRI* and *MI*) mediate the association between depression severity (CDRS) and one of the three factors *emotional dysregulation*, *relationship difficulties* and *nssi* (IED 27 SR factors). The parallel mediation models confirmed a significant association between depression severity and EF impairment for all three factors (see Figure 2 for the factor *emotional dysregulation* (total effect *c*: β = 0.0453, *p* < 0.001), Figure 3 for the factor *relationship difficulties* (total effect *c*: β = 0.0351, *p* < 0.001) and Figure 4 for the factor *nssi* (total effect *c*: β = 0.0423, *p* < 0.001)). After entering the mediators *BRI* and *MI* into the parallel mediation models, depression severity predicted both mediators significantly, *BRI*: β = 0.3103, *p* = 0.006, *MI*: β = 0.3506, *p* = 0.012 (same values for all three models). The *BRI* predicted *emotional dysregulation* significantly, β = 0.0165, *p* = 0.011; *while* the *MI* did not, β = 0.0037, *p* = 0.467. We found that the relationship between depression severity and *emotional dysregulation* is mediated by the *BRI* (95% CI [0.0007–0.0118]), but not the *MI* (95% CI [−0.0020–0.0061]), (Figure 2). Similarly, *relationship difficulties* were predicted significantly by the *BRI*, β = 0.0392, *p* < 0.001, but not the *MI*: β = 0.0024, *p* = 0.581. We found that the relationship between depression severity and *relationship difficulties* is mediated by the BRI (95% CI [0.0036–0.0215]) but not the MI (95% CI [−0.0019–0.0048]) (Figure 3). In contrast, neither the *BRI* (95% CI [−0.0043–0.0050]) nor the *MI* (95% CI [−0.0057–0.0033]) significantly mediated the relationship between depression severity and *nssi* (Figure 4).

## Discussion

The present study investigated the deficits of EF in daily life in moderately to severely depressed adolescents and whether the observed deficits in EF are related to borderline personality features. Furthermore, we examined the agreement between self- and parent-rated deficits in EF.

### Executive functioning in clinically depressed adolescents

Overall, none of the BRIEF scale scores lay above T > 65 indicating clinical impairment, neither for the self-rated nor for the parent-rated scales of the present sample. The self-rated scales *Emotional Control, Working Memory*, and *Plan/Organize*, as well as the index *MI*, had a mean score above T > 60, which might indicate subclinical deficits. On average, the deficits seem to be subtle and more cognitive rather than behavioural, which is reflected by the high score of the *Metacognition Ind*ex. In the present sample, parents did not rate their children as clinically impaired, as none of the scales scored within the subclinical or clinical range. This is in contrast to the study by Vesco et al. (2018), who found the *MI* and the *BRI* in the clinical range according to the parents’ ratings in a sample of 95 depressed children with a mean age of 11 years. This difference may be explained by the fact that their sample was mixed, with children presenting depressive or bipolar disorder and a high comorbidity with ADHD (61%). In the study by Schmid and Hammar (2021) the *MI* was in a clinical range in the group of patients who still had depressive symptoms one year after the onset of the first MDD episode. Unfortunately, the scores from the first episodes were not published.

The adolescents in our sample reported the greatest T-values for the scale *Working Memory*. Deficits in working memory in adolescent patients with pMDD compared to healthy controls have been reported in previous studies (Baune et al., 2014; Friedman et al., 2018), and working memory seems to be one of the EF most strongly affected by depression. While deficits in other EF have been reported in several studies with adult MDD patients based on objective tests (Stordal et al., 2004; Wagner et al., 2012). Vilgis et al. (2015) concluded in their systematic review that there is little support for deficits in EF in pMDD in minors. Our results seem to corroborate this conclusion given that only the adolescents themselves described subtle EF deficits in some of the cognitive scales.

### Comparison and agreement of self-rated and parent-rated EF

Overall, the adolescents in this sample reported significantly stronger deficits in EF than their parents, especially on the BRIEF scales *Inhibit, Emotional Control, Working Memory, Plan/Organize*, and *Organization of Materials*. The difference between the self-perceived and the parents’ EF ratings is also reflected in the poor agreement between the two reports. This is in contrast to the results of Egan et al. (2018), who compared the BRIEF self- and parent-rated scores in a community sample. There are at least two possible explanations for this discrepancy; first, adolescents with pMDD might overestimate their deficits because they have a biased perception due to the pMDD symptomology (Dozois et al., 2012; Jónsdóttir et al., 2021). Second, parents are potentially underestimating the deficits in their children. Orchard et al. (2019) found that parents of adolescents with subthreshold depression struggle to observe subtler cognitive deficits. However, the present study’s sample consists of adolescents with more pronounced pMDD symptoms. Therefore, it is reasonable to assume that parents may underestimate deficits in their children, as suggested by Upton et al. (2008). This underestimation may be due to a lack of awareness or understanding of their children’s experiences or even a tendency to minimize or deny the presence of issues to avoid confronting them. Recognizing the discrepancies between self-ratings and parent-ratings of EF deficits can help clinicians identify potential biases and better interpret the assessments. This understanding can lead to more accurate diagnoses and personalized treatment plans. Furthermore, incorporating both self-ratings and parent-ratings into the assessment process can provide a more comprehensive picture of the adolescent’s functioning, allowing clinicians to address areas of concern that may otherwise go unnoticed. Involving both adolescents and parents in the treatment process can also foster open communication, enhance therapeutic alliance, and improve treatment adherence and outcomes.

### Associations between EF and BPF

To investigate the possible overlap between BPF and EF, we conducted two correlation analyses, one with BRIEF SR scales and IED-27 SR factors and one with BRIEF P scales and IED-27 P factors. The analysis revealed that the BRIEF scales *Inhibit* and *Emotional Control* correlated strongly with the IED-27 *relationship difficulties* factor for parent- and self-rating. The scales of the *MI*, such as *Working Memory, Organisation of Materials*, and *Monitor*, did not correlate as highly, but were nevertheless still higher when based on the adolescents’ self-ratings. Very similar results were obtained when we used BRIEF 2 scales and indices (Gioia et al., 2015) (Supplementary Tables 3, 4), suggesting that the present findings are not dependent on the original BRIEF two-factor structure.

Some of the behaviors that are characteristic of BPF, such as *emotional instability*, were found to be related to EF deficits. This is also reflected in the fact that the questionnaires contain similar items (see supplement Supplementary Table 1 for IED-27 SR items). For example, the IED-27 SR item “My feelings went up and down like a roller coaster” (Dreysse et al., 2020) is similar to the BRIEF SR item on the *Emotional Control* scale “mood changes rapidly”. Therefore, it is somewhat surprising that the BRIEF scale *Emotional Control* showed the strongest correlation with the IED-27 *relationship difficulties* factor and not with the *emotional dysregulation* factor. Perhaps it is in relationship difficulties that deficits in EF may be observed best as they come into light in social interactions. The IED-27 SR *emotional dysregulation* factor also correlated with *Inhibit* and *Emotional Control*, but only moderately. Thus, this typical BPF of emotional dysregulation is only partly attributable to a perceived deficit in the executive function of *Emotional Control* and might encompass further aspects that are not related to EF deficits per se. The mediation models support these findings, as the *BRI* significantly mediated the relationship between depression severity and the factors *emotional dysregulation* and *relationship difficulties* for self-rating. Similar to the results of Wante et al. (2017), who defined emotion regulation strategies as a mediator between EF impairment and depressive symptoms; however, adolescents in our sample have a clinically diagnosed pMDD.

The *self-injuries and suicidal behavior* factor of the IED-27, which is a symptom of both pMDD and BPD, did not correlate with any of the BRIEF scales, either in parent-ratings or in self-ratings. In the mediation model, although depression severity predicted the level of *nssi*, this relationship was not mediated by neither of the BRIEF indices. In contrast to our findings, Fikke et al. (2011) reported working memory deficits in adolescents with high-severity non-suicidal self-injury (NSSI) and impaired inhibitory control in adolescents with low-severity NSSI compared with healthy controls. Other studies linked suicidality to impaired decision-making in adolescents (Bridge et al., 2012) and adults (Allen et al., 2019). In adults, the most consistent findings of deficits in EF were found for suicide attempters with depression (Lara et al., 2015; Lalovic et al., 2022). However, one study reported that adolescents at risk of suicide did not show any EF deficits but rather impairments in other neurocognitive domains, such as complex cognition, episodic memory, or social cognition (Ortuño-Sierra et al., 2020). As in our sample, suicidal and non-suicidal self-harm behaviour appeared to be unrelated to perceived EF deficits.

### Depression severity and impaired EF as predictors for BPF

For self- and parent-rated models, depression severity was the strongest predictor of elevated BPF in our sample, highlighting the high comorbidity rate of pMDD and BPD (Pham-Scottez, 2016). Furthermore, the *Emotional Control* (parent-rating) and *Inhibit* (self-rating) scales also significantly predicted BPF. For parents, emotional control might be one of the most discerning executive functions and it is associated with emotional instability as well as relationship difficulties. Interestingly, in the self-rated version, inhibit was the EF scale most strongly associated with BPF. More so, the BRI, which includes the *Inhibt* subscale, significantly mediated the relationship between depression severity, and emotional dysregulation and relationship difficulties. This suggests that problems with inhibition may be one of the mechanisms leading to the typical symptoms of BPD in depressed adolescents. Ernst et al. (2018) found a general inhibitory dysfunction in adult patients with MDD and BPD compared to those with MDD only.

The mediation analysis suggests that depression symptoms could lead to deficits in executive functions related to behavioral control, which in turn negatively affect emotional regulation and lead to problems in social relationships. Allan et al. (2016) argued in a review article that there is a positive feedback loop between EF and health-related behaviour. Accordingly, depressive symptoms might be related to deficits in executive functioning and BPF in a negative feedback loop. For example, problems in relationships with others might exacerbate depressive symptoms such as guilt and self-worth, which increases the severity of depression which in turn negatively affects EF. According to a recent review, EF in MDD is related to theory of mind (ToM) (Pagnoni et al., 2022). Deficits in ToM could hinder accurate categorization of another person’s mental state and lead to relationship problems.

Recognizing the relationship between pMDD, BPF, and EF can help clinicians develop a more nuanced understanding of the factors contributing to an adolescent’s psychopathology. This insight can guide the selection of targeted interventions for at-risk adolescents, such as cognitive-behavioral therapy (CBT) that addresses both emotion regulation and executive function skills. By focusing treatment on the overlapping features of pMDD and BPF and targeting the underlying cognitive and emotional processes involved in both, clinicians may be able to provide more effective interventions (Basharpoor et al., 2022). This in turn can lead to improvements in patient well-being and quality of life by enhancing overall psychosocial functioning in adolescents with comorbid BPF.

## Limitations

The main limitation of our analysis lies in the sample. We assessed deficits in EF in a sample of adolescents diagnosed with pMDD with moderate to severe symptom severity and assessed the impact of BPF, without diagnosing BPD itself. Thus, it remains unclear whether these results might also apply to manifest BPDs. Furthermore, our sample might be somewhat biased due to the inclusion criteria of the Omega-3-pMDD Study, such as the exclusion of patients with substance dependency (Sansone and Sansone, 2011). This might have contributed to the imbalance between girls and boys in this sample, which was about 3:1. Nevertheless, this girl-boy ratio is in accordance with most of the literature about pMDD, 3:1 (Bernaras et al., 2019). In addition, EF were assessed only using rating scales and was not based on objective measures.

## Conclusion

To summarize, our data show that adolescents with depression generally do not perceive their EF as clinically impaired. However, adolescents who perceive greater deficits in EF in their daily life also report higher borderline personality features. Parents confirmed the relationship between EF deficits and greater BPF but did not report the EF deficits to be as high as the adolescents themselves. It is difficult to determine whether the perceived deficits are present but not perceived by the parents or related to a deficit in self-perception in adolescents with elevated BPF. Depression is characterized by a negative bias in self-perception, and these negative distortions in perception might be aggravated by comorbid BPF, likely leading to an overestimation of deficits in EF in daily life. Even more so, impairment in certain executive functions, especially in the executive functions related to behavioural control, might be related to borderline personality features, such as emotional dysregulation and relationship difficulties. EF have a strong impact on various aspects of daily life and intact executive functioning is crucial for succeeding in school and early professional development. Deficits in these executive functions might lead to problems typically associated with borderline personality disorder, further affecting psychosocial functioning and possibly reinforcing depressive symptoms in a negative feedback loop. Therefore, the assessment and treatment of deficits in executive functioning in adolescents with depression might have a positive impact on overall symptomatology in this highly affected patient group.

## Data availability statement

The raw data of this study will be made available by the authors upon request.

## Ethics statement

The studies involving human participants were reviewed and approved by Kantonale Ethikkommission Zürich (KEK): Lead-Ethic Commission, Ethikkommission Ostschweiz (EKOS), Ethikkommission Nordwest- und Zentralschweiz (EKNZ), number: 2016-02116. Written informed consent to participate in this study was provided by the participants’ legal guardian/next of kin and the study participants.

## Author contributions

MA and IH: analysis and interpretation of the data. MA, IH, SoE, and GB: writing, editing, and revision of the manuscript. KS, SoE, SB, LW, UM-K, BC-W, and BR: Resources. GB: Funding acquisition. All authors have read and agreed to the published version of the manuscript.

## The Omega-3 Study Team

The Omega-3 Study Team contributed with implementation of the design with following roles: Sponsor-investigator of the trial is GB (Department of Child and Adolescent Psychiatry, University Hospital of Psychiatry, University of Zurich, Neumünsterallee 9, 8032 Zurich, Switzerland; gregor.berger@puk.zh.ch; +4143 499 26 26). Chief investigator is KS. IH is study coordinator. Principal investigators and research psychologist from the clinical sites are as follows: Research psychologists: Noemi Baumgartner, Sophie Emery, Mona Albermann, and Kristin Nalani (Department of Child and Adolescent Psychiatry, University Hospital of Zurich); Principal Investigator Basel: KS; Investigators and research psychologists: Oliver Pick, Alain Di Gallo, and Michael Strumberger (Department of Child and Adolescent Psychiatry, Psychiatric University Hospitals Basel); Principal Investigator Basel-Stadt: Brigitte Contin; Investigator: Stefan Müller (Child and Adolescent Psychiatric Services Baselland); Principal Investigator: Silke Bachmann and Lars Wöckel, Investigator: Simone Heitzer (Clienia Littenheid); Principal Investigator: Bruno Rhiner; Investigators: Amir Yamini (Child and Adolescent Psychiatric Services Thurgau); Principal Investigator: Suzanne Erb; Investigators: Michael Schmid (Child and Adolescent Psychiatric Services St. Gallen); Principal Investigator: Ulrich Müller-Knapp; Investigator: Ioannis Christodoulakis (Klinik Sonnenhof). Ulrike Held and Burkhardt Seifert (retired) are statistical consultants. Edna Grünblatt is head of the department for translational molecular psychiatry (Department of Child and Adolescent Psychiatry, University Hospital of Zurich). Martin Hersberger is head of the division of Clinical Chemistry and Biochemistry at the University Children’s Hospital Zürich and his Ph.D student Ivan Hartling of the division of Clinical Chemistry and Biochemistry who will analyze the bioactive lipids; Romuald Brunner (University of Heidelberg), Jürgen Drewe (University of Basel), and Julia Braun (Epidemiology, Biostatistics, and Prevention Institute, University of Zürich) are members of the Data Monitoring Committee. Jenny Peterson, Clinical Trials Pharmacy (Kantonsapotheke) Zürich, responsible for the packaging, handling, and quality of the study medication.
