# Supplementary material for: Executive functions and borderline personality features in adolescents with major depressive disorder
Source: Front Hum Neurosci. 2023 Jun 22;17:957753. doi: 10.3389/fnhum.2023.957753 (PMC10325791; doi:10.3389/fnhum.2023.957753)
Supplement: Supplementary file 1 [file Data_Sheet_1.pdf]

Supplementary Material

Table 1

Correlations IED-27 self-rating (SR) with BRIEF SR scales and IED-27 factors – raw scores

IED-27 SR items	BRIEF SR scales										BRIEF indicies	
	Inhibit	Shift	Behavioral Shift	Cognitive Shift	Emotional Control	Monitor	Working Memory	Plan/ Organize	Organization of Materials	Task Completion	Behavioral Regulation Index (BRI)	Meta-cognition Index (MI)
1. I was afraid of losing control over my feelings.	.318***	.235**	.260**	.213*	.298**	.285***	.253**	.268^{mc}	.200*	.208*	.356***	.267^{mc}
2. I hurt myself by intentionally bunking my head, arm, or other bodily parts.	.244**	.156	.134	.113	.128	.280***	.209*	.207*	.239**	.115	.242**	.216**
3. I hurt myself by superficial cutting or scratching.	.163	-.039	.003	-.068	.051	.215**	.078	.105	.157	-.045	.111	.079
4. I was afraid of losing control over my actions.	.249**	.151	.147	.163	.224**	.182*	.165*	.178*	.200*	.122	.258**	.187*
5. I was so angry that I could hardly control myself.	**.553****	.405***	.368***	.371***	**.575*****	.345***	.266^{mc}	.267^{mc}	.245**	.165*	**.611*****	.268^{mc}
6. I had eating attacks.	.292**	.284***	.238**	.309***	.444***	.159	.238**	.298***	.258**	.248**	.389***	.296***
7. I prepared to attempt suicide.	-.126	-.133	-.048	-.189*	.002	-.040	-.140	-.135	-.105	-.209*	-.098	-.169*
8. My feelings went up and down like a roller coaster.	.333***	.241**	.239**	.208*	.367***	.317***	.206*	.240**	.264^{mc}	.183*	.394***	.250**
9. I acted very erratically.	.420***	.192*	.142	.241**	.303***	.241**	.261**	.325***	.337***	.160	.378***	.303***
10. I argued.	.325***	.237**	.165*	.273**	.433***	.251**	.267^{mc}	.393***	.366***	.244**	.401***	.358***
11. I had phantasies of vengeance.	.126	.059	.080	-.008	.125	.214*	.168*	.182*	.182*	.175*	.152	.200*
12. I was thinking of ways to kill myself.	-.011	-.050	.023	-.099	.099	.016	.029	.009	-.001	-.118	.018	-.023
13. My feelings changed rapidly between bad temper, anger, fear, loneliness, and sadness.	.342***	.388***	.348***	.351***	.408***	.311***	.298***	.308***	.273***	.271**	.456***	.328***
14. My relationships were constantly up and down.	.278**	.122	.046	.216**	.332***	.184*	.173*	.303***	.291**	.136	.299***	.253**
15. I did something without considering the consequences for me or for someone else.	.432***	.235**	.209*	.240**	.389***	.414***	.270^{mc}	.279***	.328***	.182*	.457***	.296***
16. I thought of killing myself.	.025	.002	.054	-.052	.027	.121	.142	.089	.087	.010	.042	.094
17. I experienced intense hatred.	.257**	.229**	.289**	.069	.241**	.228**	.232**	.157	.145	.118	.299***	.187*
18. I hurt myself by burning.	.085	-.164*	-.150	-.093	.054	-.030	-.022	-.062	-.016	-.121	-.002	-.065

19. I was overwhelmed by my feelings.	.306***	.241**	.277***	.185*	.337***	.187*	.176*	.241**	.175*	.151	.349***	.213
20. I was very tense and under high pressure.	.265 mc	.255**	.290***	.143	.246**	.250**	.227**	.159	.221**	.190*	.316***	.224**
21. I hurt another person's feelings, which I regretted afterwards.	.184*	.162	.105	.190*	.244**	.190*	.210*	.226**	.187*	.166*	.244**	.225**
22. I was involved in trouble with other people.	.319***	.124	.088	.175*	.289***	.278***	.197*	.294**	.269 mc	.231**	.319***	.280***
23. I consoled myself with fantasies of suicide.	.092	.079	.139	-.055	.119	.126	.132	.068	.079	-.004	.126	.078
24. My emotions were muddled.	.286***	.236**	.247**	.212*	.372***	.222**	.182*	.262**	.224**	.160	.358***	.235**
25. I hurt myself by deep cuttings.	.000	-.159	-.186*	-.055	-.063	-.010	.036	.005	.097	-.031	-.073	.025
26. I was angry.	.487***	.319***	.224**	.360***	**.529*****	.312**	.255**	.257**	.329***	.203*	**.537*****	.291***
27. I vomited.	-.009	-.036	.009	-.068	.154	.006	-.081	.035	.055	-.083	.041	-.026
Emotional dysregulation	.407***	.319***	.330***	.263**	.412***	.328***	.294***	.314***	.298***	.230**	.466***	.322***
Relationship difficulties	**.595*****	.312***	.241**	.342***	**.551*****	.431***	.339***	.415***	.417***	.280 mc	**.570*****	.408***
Self-injuries and suicidal behavior	.092	-.007	.045	-.068	.081	.149	.113	.083	.114	-.040	.088	.074

Note. N = 144. English items from Dreysse et al. (2020). Starting all with "During the past month…". Bold = strong effects. IED-27 factors from Dreysse et al. (2021). SR = self-rating.
*** $p < .001$. ** $p < .01$. * $p < .05$. mc < .002 (corrected for multiple comparisons: 0.05/30).

Table 2

Correlations IED-27 parent-rating (P) with BRIEF P scales - sorted by IED-27 factors parent-rating – raw scores

IED-27 P items	BRIEF P scales										BRIEF Indices	
	Inhibit	Shift	Emotional Control	Initiate	Working-Memory	Plan/ Organize	Organization of Materials	Monitor	Task-monitor	Self-monitor	Behavioral Regulation Index (BRI)	Meta-cognition Index (MI)
**Emotional dysregulation**	.167*	.325***	.433***	.225**	.255**	.226**	.239**	.149	.105	.144	.392***	.264**
1. I was afraid that my child would lose control of his/her emotions.	.100	.193*	.205*	.098	.169*	.118	.131	.077	.025	.106	.207*	.150
4. I was afraid that my child would lose control over her/his actions.	.148	.111	.200*	.096	.127	.136	.140	.050	.057	.024	.194*	.139
8. The feelings of my child went up and down like a roller coaster.	.027	.239**	.338***	.134	.175*	.113	.214*	-.006	.017	-.029	.262**	.158

9. My child acted very erratically.	.269 mc	.300***	.416***	.190*	.199*	.237**	.293**	.187*	.121	.192*	.414**	.271 mc
13. My child's feelings changed rapidly between bad temper, anger, fear, loneliness, and sadness.	.092	.194*	.346***	.138	.173*	.131	.115	.038	.002	.065	.274**	.152
17. My child experienced intense hatred.	.157	.309***	.281***	.279***	.099	.174*	-.003	.228**	.143	.241**	.308**	.186*
19. My child was overwhelmed by her/his feelings.	.062	.172*	.312***	.176*	.190*	.158	.243**	.122	.148	.049	.239**	.216
20. My child was very tense and under high pressure.	.060	.218**	.182*	.098	.089	.165*	.064	.135	.075	.153	.190*	.140
24. My child's emotions were muddled.	.031	.157	.240**	.086	.092	.086	.205*	.028	.017	.030	.186*	.120
**Relationship difficulties**	.454***	.416***	**.552*****	.342***	.242**	.322***	.151	.366***	.214*	.402***	**.593*****	.348***
5. My child was so angry that she/he could hardly control her-/himself…	.351***	.246**	**.557*****	.282***	.176**	.253**	.100	.183*	.111	.197*	.496***	.248**
10. He/she argued.	.307***	.166*	.290***	.146	.051	.170*	-.007	.174*	.080	.217**	.319***	.133
11. My child had phantasies of vengeance.	.203*	.129	.164*	.082	-.009	.124	-.002	.135	.011	.225**	.204*	.081
14. Her/his relationships were constantly up and down.	.176*	.305***	.335***	.174*	.133	.137	.185*	.174*	.061	.237**	.340***	.191*
15. My child did something without considering the consequences for me or for someone else.	.300***	.168*	.201*	.251**	.303***	.235**	.226**	.321***	.260**	.273***	.273***	.325***
21. She/he hurt another person's feelings, which she/he regretted afterwards.	.254**	.192*	.118	.059	.118	.092	.088	.244**	.101	.314**	.233**	.142
22. My child was involved in trouble with other people.	.304***	.342***	.385***	.203*	.197*	.261**	-.026	.311***	.192*	.331***	.427***	.239**
26. My child was angry.	.203*	.354***	.429***	.313***	.083	.177*	.087	.140	.130	.102	.414***	.191*
**Self-injuries and suicidal behavior**	.089	.086	.103	.126	.108	.055	.157	.068	.125	-.020	.116	.120
2. My child did injure her-/himself by intentionally bunking her/his head, arm or other bodily parts.	.159	.113	.089	.158	.156	.184*	.022	.160	.131	.135	.144	.173*
3. My child did hurt her-/himself by superficial cutting or scratching.	.070	.032	.006	.152	.175*	.048	.062	.091	.077	.074	.040	.127

7. My child prepared to attempt suicide.	-.061	-.023	.035	-.062	-.094	-.062	.060	-.113	.003	-.200*	-.014	-.069
12. My child was thinking of ways to kill her-/himself.	.132	.079	.162	.107	.090	.084	.164*	.083	.153	-.025	.158	.125
16. She/he thought of killing her-/himself.	.056	.081	.164*	.087	.054	.040	.141	.071	.152	-.044	.131	.089
23. My child consoled her-/himself with fantasies of suicide.	.083	.092	.089	.085	.005	-.002	.180*	.077	.146	-.028	.108	.070
25. My child hurt her-/himself by deep cuttings.	.028	.085	-.022	.122	.165*	.012	.164*	-.020	-.028	-.004	.030	.106
**Excluded items:**												
6. My child had eating attacks.	.119	.161	.205*	.127	.066	.115	.232**	.109	.074	.109	.203*	.152
18. She/she hurt her-/himself by burning.	-.008	.040	.070	.141	.072	.027	.064	.056	.090	-.002	.046	.082
27. She/he vomited.	-.006	-.177*	-.143	-.045	-.159	-.122	.111	-.058	-.034	-.065	-.135	-.083

Note. N = 144. Self-rating items from Dreysse et al. (2020), translated from German to English by the authors. Starting all with "During the past month…". Bold = strong effects. IED-27 factors from Dreysse et al. (2021).
***p < .01. p < .01. * p < .05. ^{mc} < .002 (corrected for multiple comparisons: 0.05/30).

Table 3

Correlations self-rating: BRIEF 2 x IED-27 SR factors – raw scores

IED-27 factors	BRIEF 2 SR scales							BRIEF 2 SR Indices		
	Inhibit	Shift	Emotional Control	Self - monitor	Working Memory	Plan/ organize	Task-Completion	Behavioral Regulation Index (BRI)	Emotional Regulation Index (ERI)	Cognitive Regulation Index (CRI)
Emotional dysregulation	.345***	.261 ^{mc}	.384***	.306***	.197*	.281***	.181*	.376***	.366***	.249 ^{mc}
Relationship difficulties	**.545*****	.384***	**.613*****	.440***	.247 ^{mc}	.397***	.227 ^{mc}	**.564*****	**.564*****	.340***
Self-injuries and suicidal behavior	.057	-.015	.058	.149	.095	.101	-.055	.012	.022	.055

Note. N = 144. Bold = strong effects. IED-27 factors from Dreysse et al. (2021). SR = self-rating.
***p < .001. ** p < .01. * p < .05. ^{mc} < .017 (corrected for multiple comparisons: 0.05/3).

Table 4

Correlations parent-ratings: BRIEF 2 P x IED-27 P Factors – raw scores

IED-27 factors	BRIEF 2 P scales									BRIEF 2 Indices		
	Inhibit	Shift	Emotional Control	Initiate	Working Memory	Plan/ Organize	Organziation of Materials	Task-Monitor	Self-Monitor	Behavioral Regulation Index (BRI)	Emotional Regulation Index (ERI)	Cognitive Regulation Index (CRI)
Emotional dysregulation	.244**	.284**	.446**	.212**	.246 mc	.257**	.226**	.088	.144	.231**	.422**	.251 mc
Relationship difficulties	.460**	.400**	**.579****	.310 mc	.266**	.307**	.272**	.212 mc	.402 mc	.492**	**.564****	.324 mc
Self-injuries and suicidal behavior	.099	.083	.130	.103	.125	.043	.118	.101	-.020	.060	.123	.116

Note. N = 144. Bold = strong effects. IED-27 factors from Dreysse et al. (2021). P = parent-rating.
** $p < .01$. * $p < .05$. $^{mc} < .017$ (corrected for multiple comparisons: 0.05/3).
